# Miliary tuberculosis with positive acid-fast bacilli in a pediatric patient

**DOI:** 10.1590/S1516-31802003000300008

**Published:** 2003-05-01

**Authors:** Sara Regina Castanheira Fernandes, Marcia Noriko de Oliveira Homa, Aghata Igarashi, Andréa Luiza Mendes Salles, Ana Paula Jaloretto, Maria Silvia Freitas, Paulo Cesar Koch Nogueira

**Keywords:** Miliary tuberculosis, Laboratory techniques and procedures, Tuberculose miliar, Técnicas e procedimentos de Laboratório

## Abstract

**CONTEXT:**

Tuberculosis is an important public health issue. The Brazilian government reported 78,460 new cases in 1999. Miliary tuberculosis is a severe form of this disease.

**OBJECTIVE:**

To report on an uncommon clinical presentation of miliary tuberculosis in a child.

**CASE REPORT:**

A 5-year old boy presented in the emergency room with fatigue and weight loss. He had had Staphylococcus aureus pneumonia 7 months before. Chest radiography revealed lobar consolidation and miliary pattern associated with small cavities in both upper lobes. Antibiotic therapy was started. The sputum was positive for acid-fast bacilli and hence the treatment recommended for tuberculosis (rifampicin, isoniazid [INH], pyrazinamide) was started. The patient was treated for 9 months and at the end of the follow-up period he had made a complete clinical recovery.

**CONCLUSION:**

Although in some particular cases sputum can be positive for acid-fast bacilli in children, limitations to the sputum test have forced pediatricians to base tuberculosis diagnosis on epidemiological data, clinical findings and radio-graphic pattern. In this particular case, we hypothesize that the sputum bacillus test was positive because *bacilli* grew inside residual pneumatoceles that were produced during previous pneumonia.

## RESUMO

**CONTEXTO:**

A tuberculose representa importante problema de Saúde Pública no Mundo. O governo brasileiro relatou 78.460 mil novos casos no ano de 1999. A tuberculose miliar é uma forma grave desta doença.

**OBJETIVO:**

Reportar uma forma atípica de manifestação da tuberculose miliar em paciente pediátrico.

**RELATO DO CASO:**

Paciente masculino, cinco anos, compareceu ao serviço de emergência com fadiga e emagrecimento importantes. A radiografia torácica tinha como resultado condensação lobar com padrão miliar associado a cavitações em lobos superiores. O paciente havia apresentado, sete meses antes, um episódio de pneumonia estafilocócia complicada com derrame pleural. A antibióticoterapia foi iniciada. O escarro da criança veio positivo para pesquisa do bacilo álcool-ácido-resistente e, portanto, o tratamento recomendado para tuberculose foi iniciado. O paciente foi tratado por nove meses e, ao fim do seguimento, apresentou recuperação clínica completa.

**CONCLUSÃO:**

Limitações do exame de escarro forçam os pediatras a investigar a tuberculose através de dados epidemiológicos, achados clínicos e do padrão miliar ao raio-x, embora, em alguns raros casos, o escarro possa ser positivo em crianças. Neste paciente, a hipótese mais provável para explicar este achado é de que os bacilos usaram as cicatrizes da pneumonia estafilocócica prévia (pneumatoceles) para alcançar concentrações expressivas no escarro e, com isso, tornar positivo o resultado do exame.

## INTRODUCTION

Tuberculosis represents a public health problem with worldwide incidence that has been increasing since 1980.^1^ This disease was under control until the beginning of the aids (human immunodeficiency virus, HIV, infection) epidemics. The Brazilian government reported 78,460 new cases of tuberculosis in 1999.^1^

Miliary tuberculosis is a severe form in which the host immunological response is insufficient and disseminated disease occurs.^1^ This paper reports an unusual laboratory finding of this disease in a pediatric patient.

## CASE REPORT

W.A.S., a 5-year-old boy, was brought to the emergency room complaining of fatigue, weight loss, and cough with purulent sputum. Fever had been noted for one week. The patient had a history of *Staphylococcus aureus* pneumonia, with a pyopneumothorax complication 7 months earlier. The child's immunization record was complete, including Bacillus Calmette-Guérin for tuberculosis (BCG).

Upon admission the child had pallid skin, dehydration, dyspnea, and fever (38° C). Examination of the respiratory system revealed reduced breathing sounds with crackles in both lungs. Abdominal examination showed no organomegaly. There was a purulent secretion in the child's right ear. Bulging of the tympanic membranes was noted.

**Figure 1:**
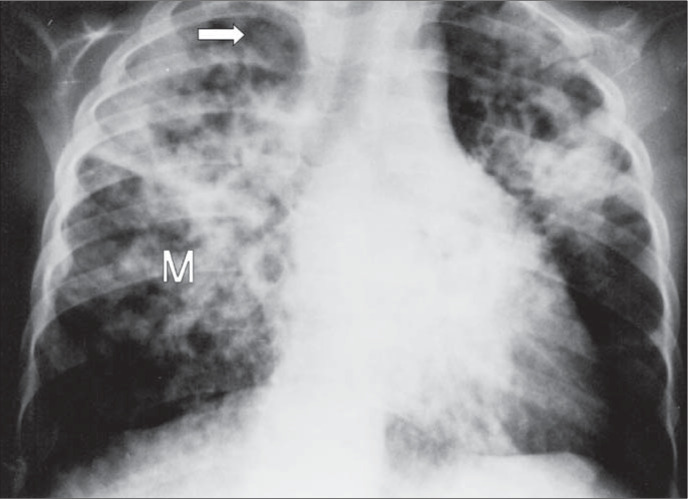
Chest x-ray upon admition. M = miliary pattern. Arrow shows cavities at the right upper lobe.

Chest X-ray revealed lobar consolidation, perihilar infiltrate, and miliary pattern associated with small cavities in both upper lobes (Figure 1). A diagnosis of pneumonia, respiratory distress and acute suppurative otitis was made. Miliary tuberculosis was suspected as a secondary hypothesis. Antibiotic therapy with ceftriaxone was started.

Purified Protein Derivative resulted in strong conversion, and the sputum smear was positive to acid-fast bacilli. The patient was isolated, and treatment for tuberculosis was started. Two serology tests for ELISA were negative.

**Figure 2:**
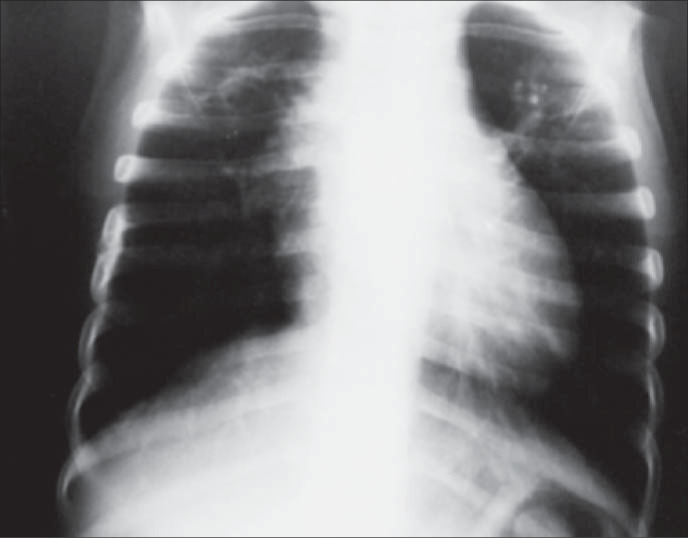
Chest X-ray within 9 months of therapy. Residual emphysema and lack of miliary lesions.

The child was discharged after 29 days of hospitalization with fortnightly follow-up for the first 2 months and monthly thereafter. After six months of therapy, he still had crackles upon physical examination. It was decided to extend tuberculosis medication for 3 additional months. Three months later, the patient presented a complete clinical recovery. Chest x-ray showed a residual emphysematous pattern (Figure 2).

## DISCUSSION

Miliary tuberculosis is a severe manifestation of *Mycobacterium* tuberculosis infection*.* It accounts for 3-7% of all reported cases of tuberculosis. Mortality can be as high as 25%, especially in infants.^2^

Systemic involvement in patients with miliary tuberculosis results in a variety of clinical presentations for this type of tuberculosis. Usually the initial manifestations are unspecific. Fever is the most common symptom (94%). This is followed by malnutrition (84.1%), adenopathy (79.3%), cough (68%), weight loss (57%), respiratory complaints (57%) and hepatosplenomegaly (44.4%).^3^

Hemoptysis seldom occurs. Bronchial constriction due to adenopathy may result in wheezes and atelectasis.^1,2^ Miliary tuberculosis must be considered in the differential diagnosis when antibiotic therapy for common organisms fails to treat pneumonia.^3^

About 50-90% of all patients present the miliary pattern with disseminated tuberculosis.^2,3^ Other radiological findings include cavities (rare) and pleural or pericardial effusions.^3^

In adults with suspected tuberculosis, the sputum smear is a helpful test. On the other hand, the diagnosis of tuberculosis in children is challenging. It requires a history of prior patient contact, together with positive sputum, clinical findings and chest x-ray. Additional useful information can be provided by the purified protein derivative, which can show whether the patient has had contact with the *bacilli*.^2,3^ The purified protein derivative shows whether or not the patient has had contact with the *bacilli*. It has low specificity and sensitivity.^1,4^ Serology for tuberculosis was developed at the beginning of the HIV epidemic because of the need for immediate treatment for those immunocom-promised patients. However, this test has a high cost, and is only used for research.^1^

For a positive acid-fast *bacilli* sputum test, at least 5,000 to 10,000 *bacilli*/millimeter of specimen are required.^1^ *Mycobacterium* tuberculosis culture needs 10-100 microorganisms.^2^

Patients with miliary tuberculosis usually do not have cavities. Therefore, they present a very low concentration of *bacilli* when compared to those with pulmonary tuberculosis, i.e. with cavities. Moreover, children do not produce as much sputum as adults.^3^

Gastric aspiration can be used to obtain specimens of swallowed sputum. Although it is uncomfortable, it is more cost-effective and less invasive than bronchoscopy. It is the best way to obtain sputum specimens from infants and some young children. Gastric aspiration to obtain specimens from children should be done in the morning, before the patient gets out of bed or eats.^1^

In Brazil, according to the Ministry of Health, the recommended treatment for extrapulmonary tuberculosis is the same as for pulmonary disease.^4^ Recent studies have shown that 6-9 months of therapy has good results.^1^

## CONCLUSION

Tuberculosis is an important reemerging public health problem. When making differential diagnoses, it must be considered, especially in developing countries. For pediatric patients, sputum specimens are not useful in most cases, since these are rarely positive. This report shows a very unusual clinical finding of the disease: positive acid-fast *bacilli* in a child with miliary tuberculosis. The only hypothesis for explaining this finding is that the *bacilli* grew inside the residual pneumatoceles, which were produced during previous pneumonia. According to such a hypothesis, the *bacilli* used the pneumatoceles as cavities to achieve enough concentration in the sputum.
